# Supplementary material for: Economic Evaluation of Proactive PTSI Mitigation Programs for Public Safety Personnel and Frontline Healthcare Professionals: A Systematic Review and Meta-Analysis
Source: Int J Environ Res Public Health. 2025 May 21;22(5):809. doi: 10.3390/ijerph22050809 (PMC12110898; doi:10.3390/ijerph22050809)
Supplement: Supplementary file 1 [file ijerph-22-00809-s001.zip › ijerph-3591298-supplementary.pdf]

Assumptions for each paper are presented separately to facilitate ROI calculation and ensure consistency in our meta-analysis across different studies.

1 Paper-Fitzhugh et al. (2024) Reference 10

1.1 Cost Calculation

Mindfit-Cop

$$\begin{aligned}\text{Total Cost} &= 38,000\text{ (development)} + 11,239.50\text{ (participation)} + 1,599.19\text{ (support)}\\ &= 50,838.69\end{aligned}$$

$$\text{Participants} = 96$$

$$\text{Cost per participant} = \frac{50,838.69}{96} = 529.57$$

Headspace

$$\begin{aligned}\text{Total Cost} &= 12,000\text{ (license)} + 17,480.25\text{ (participation)}\\ &= 29,480.25\end{aligned}$$

$$\text{Participants} = 149$$

$$\text{Cost per participant} = \frac{29,480.25}{149} = 197.18$$

CER Comparison

Layard (2016) suggests an intervention is cost-effective if it improves life satisfaction at a CER below £2,500 per point per year.

If either Mindfit-Cop or Headspace has a negative CER, it means the intervention resulted in cost savings.

1.2 Converting Cost-Effectiveness Ratio (CER) to Return on Investment (ROI)

To convert the Cost-Effectiveness Ratio (CER) into Return on Investment (ROI) and calculate the Confidence Intervals (CI) for ROI, we use the following formula:

$$ROI = \frac{\text{Net Benefits}}{\text{Total Costs}} \times 100$$

Where:

- **Net Benefits** = Savings or increased productivity from the intervention.
- **Total Costs** = Cost per participant.

Step 1: Convert CER to Net Benefits

The Cost-Effectiveness Ratio (CER) is given as the cost per additional point of life satisfaction per year. To obtain Net Benefits, we assume:

$$\text{Net Benefits} = -\text{CER} \times \text{Life Satisfaction Improvement}$$

For simplicity, we assume a 1-point life satisfaction improvement per year (as per Layard's threshold). If CER is negative, the intervention leads to savings, and net benefits are positive.

Step 2: Compute ROI

Using:

$$ROI = \frac{-CER}{\text{Cost per Participant}} \times 100$$

where:

- Cost per participant for **Headspace**: £197.85
- Cost per participant for **Mindfit-Cop**: £529.57

Step 3: Compute Confidence Intervals (CI) for ROI

Since CER has a 95% CI, we compute ROI for both the upper and lower bounds of each CI.

1.3 Headspace: ROI and Confidence Intervals

**Scenario**	**CER (£)**	**95% CI**	**ROI (%)**	**95% CI for ROI (%)**
No change in productivity	223.37	(161.18 to 363.71)	-112.91	(-183.86 to -81.46)
Reduction in absence days only	-27.77	(-42.22 to -20.04)	14.03	(10.13 to 21.34)
Reduction in absence + 1% productivity increase	-1,395.72	(-2,272.62 to -1,007.12)	705.16	(509.18 to 1,148.58)
Reduction in absence + 2% productivity increase	-2,676.81	(-4,358.60 to -1,931.52)	1,352.49	(976.23 to 2,202.76)
1% productivity increase (no absence reduction)	-1,144.58	(-1,863.69 to -825.90)	578.66	(417.37 to 941.75)
2% productivity increase (no absence reduction)	-2,425.67	(-3,949.67 to -1,750.30)	1,226.50	(884.58 to 1,996.77)

Table 1: ROI and Confidence Intervals for Headspace

1.4 Mindfit-Cop: ROI and Confidence Intervals

**Scenario**	**CER (£)**	**95% CI**	**ROI (%)**	**95% CI for ROI (%)**
No change in productivity	1,423.13	(644.36 to 6,822.62)	-268.72	(-1,288.94 to -121.70)
Reduction in absence days only	530.47	(240.19 to 2,543.14)	-100.19	(-480.21 to -45.38)
Reduction in absence + 1% productivity increase	-1,326.42	(-6,358.99 to -600.57)	250.48	(113.43 to 1,200.54)
Reduction in absence + 2% productivity increase	-3,028.58	(-14,519.28 to -1,371.27)	571.88	(258.91 to 2,742.74)
1% productivity increase (no absence reduction)	-433.76	(-2,079.51 to -196.40)	81.91	(37.09 to 392.63)
2% productivity increase (no absence reduction)	-2,135.92	(-10,239.79 to -967.10)	403.39	(182.66 to 1,933.93)

Table 2: ROI and Confidence Intervals for Mindfit-Cop

2 Paper Muir et al., 2022 Reference 11

2.1 ROI Calculation

$$ROI = \frac{\text{Net Savings}}{\text{Intervention Cost}} \times 100 \tag{1}$$

where:

- **Net Savings** = (Cost in Status Quo) - (Cost in Burnout Reduction Program)
- **Intervention Cost** = Cost of the Burnout Reduction Program

2.2 Step 1: Calculate Net Savings per RN

From the simulation results:

$$\begin{aligned}
\text{Status Quo Cost per RN} &= 49,373 \\
\text{Burnout Reduction Program Cost per RN} &= 40,689 \\
\text{Net Savings per RN} &= 49,373 - 40,689 = 8,684
\end{aligned}$$

2.3 Step 2: Calculate ROI

$$ROI = \frac{8,684}{40,689} \times 100 \tag{2}$$

$$ROI \approx 21.3\% \tag{3}$$

2.4 Annual ROI Calculation

Using annual turnover costs:

$$\begin{aligned}
\text{Status Quo Annual Cost per RN} &= 16,736 \\
\text{Burnout Reduction Program Annual Cost per RN} &= 11,592 \\
\text{Annual Net Savings per RN} &= 16,736 - 11,592 = 5,144 \\
\text{Annual Intervention Cost (Salary Bonus)} &= 5,627
\end{aligned}$$

$$ROI_{\text{annual}} = \frac{5,144}{5,627} \times 100 \tag{4}$$

$$ROI_{\text{annual}} \approx 91.4\% \tag{5}$$

2.5 Conclusion

- **Overall ROI:** 21.3% (for the full program per RN).
- **Annual ROI:** 91.4%, meaning the hospital recovers nearly all intervention costs within a year through reduced RN turnover.

The burnout reduction program is a high-return investment, leading to cost savings and improved RN retention.

2.6 Step 1: Identify Cost Ranges

From the simulation and sensitivity analysis:

- **Status Quo Cost per RN:** $30,000 to $150,000
- **Burnout Reduction Program Cost per RN:** $\leq 50,000$ (typically around $40,689)
- **Net Savings per RN:** Range from:

$$\begin{aligned}
(30,000 - 50,000) \quad &\text{to} \quad (150,000 - 40,689) \\
(-20,000) \quad &\text{to} \quad 109,311
\end{aligned}$$

  (Negative savings means the program costs more in that scenario.)

- **Intervention Cost:** $40,689 to $50,000

2.7 Step 2: Calculate ROI Range

$$ROI_{\text{min}} = \frac{-20,000}{50,000} \times 100 = -40\% \tag{6}$$

$$ROI_{\text{max}} = \frac{109,311}{40,689} \times 100 \approx 268.6\% \tag{7}$$

3 Paper-Moran et al., 2020 Reference 13

3.1 Return on Investment (ROI) Calculation

Return on investment (ROI) is calculated using the formula:

$$ROI = \frac{\text{Net Monetary Benefit (NMB)}}{\text{Cost of Investment}} \times 100\%$$

Given:

$$\text{Mean NMB} = \$23,360, \quad \text{95\% CI for NMB} = (-\$3,006, \$161,278)$$

$$\text{Cost of RISE per nurse} = \$656.25, \quad \text{Number of nurses} = 80$$

Mean ROI

$$ROI_{\text{mean}} = \frac{23,360}{656.25 \times 80} \times 100\%$$

$$ROI_{\text{mean}} = 44\%$$

95% Confidence Interval for ROI

Lower Bound:

$$ROI_{\text{low}} = \frac{-3,006}{656.25 \times 80} \times 100\% = -6\%$$

Upper Bound:

$$ROI_{\text{high}} = \frac{161,278}{656.25 \times 80} \times 100\% = 307\%$$

4 Paper-Wijnen et al., 2020 Reference 14

4.1 ROI and Confidence Interval Calculation

The Return on Investment (ROI) is calculated as:

$$ROI = \left(\frac{\text{Net Benefit}}{\text{Cost of Intervention}} \right) \times 100\% \tag{8}$$

Given:

- Net Benefit = €2981
- 95% Confidence Interval for Net Benefit = (-€329 to €6291)
- Cost of Intervention = €6912

The ROI is:

$$ROI = \left(\frac{2981}{6912} \right) \times 100\% = 43.15\% \tag{9}$$

For the 95% confidence interval:

$$\text{Lower Bound} = \left(\frac{-329}{6912} \right) \times 100\% = -4.76\% \tag{10}$$

$$\text{Upper Bound} = \left(\frac{6291}{6912} \right) \times 100\% = 91.06\% \tag{11}$$

5 Paper-Painter et al., 2017 Reference:15

5.1 Given Data

- **Cost of Intervention**: \$2029 per patient per year
- **Willingness-To-Pay (WTP) per QALY**: \$150,000
- **Incremental Cost-Effectiveness Ratio (ICER)**:
  - Median ICER: \$185,565 per QALY
  - Lower Bound ICER: \$57,675 per QALY
  - Upper Bound ICER: \$395,743 per QALY

5.2 QALYs Gained Calculation

QALYs gained are calculated as:

$$\text{QALYs Gained} = \frac{\text{Cost of Intervention}}{\text{ICER}} \tag{12}$$

Thus,

$$\text{QALYs (Median)} = \frac{2029}{185,565} = 0.01093$$
$$\text{QALYs (Lower Bound)} = \frac{2029}{395,743} = 0.00513$$
$$\text{QALYs (Upper Bound)} = \frac{2029}{57,675} = 0.03518$$

5.3 Net Benefit Calculation

Net Benefit is given by:

$$\text{Net Benefit} = (\text{QALYs Gained} \times \text{WTP}) - \text{Cost of Intervention} \tag{13}$$

Thus,

$$\text{Net Benefit (Median)}$$
$$\text{Net Benefit (Lower Bound)}$$
$$\text{Net Benefit (Upper Bound)} = (0.03518 \times 150,000) - 2029 = \$3248.00$$

5.4 ROI Calculation

ROI is given by:

$$ROI = \frac{\text{Net Benefit}}{\text{Cost of Intervention}} \times 100\% \tag{14}$$

Thus,

$$\text{ROI (Median)} = \frac{-380.50}{2029} \times 100\% = -18.75\%$$
$$\text{ROI (Lower Bound)} = \frac{-1289.50}{2029} \times 100\% = -63.58\%$$
$$\text{ROI (Upper Bound)} = \frac{3248.00}{2029} \times 100\% = 160.12\%$$

5.5 Confidence Interval for ROI

Based on the interquartile range of ICER, the 95% Confidence Interval for ROI is:

$$\text{ROI CI} = [-63.58\%, 160.12\%] \tag{15}$$

This indicates that while the intervention could potentially generate a positive ROI under optimistic assumptions, it is more likely to result in a financial loss under the median ICER scenario.

6 Paper-Milligan-Saville et al., 2017 Reference 16

ROI is reported as:

$$ROI = 9.98 \tag{16}$$

Thus, the ROI is **£9.98 per £1 spent**.

6.1 Standard Error (SE) for Cost Savings

We are given the confidence intervals for work-related sick leave:

- **Pre-Training Sick Leave CI**: (1.11%, 2.01%) $\Rightarrow$ SE = 0.23%
- **Post-Training Sick Leave CI**: (0.83%, 1.73%) $\Rightarrow$ SE = 0.23%

The reduction in work-related sick leave is:

$$\text{Reduction} = 1.56\% - 1.28\% = 0.28\% \tag{17}$$

Since pre- and post-training SE values are independent, we propagate the error:

$$SE_{\text{reduction}} = \sqrt{SE^2_{\text{pre}} + SE^2_{\text{post}}} \tag{18}$$

$$SE_{\text{reduction}} = \sqrt{(0.23\%)^2 + (0.23\%)^2} = \sqrt{0.000529 + 0.000529} \tag{19}$$

$$SE_{\text{reduction}} = \sqrt{0.001058} = 0.033\% \approx 0.33\% \tag{20}$$

Now, using the cost of work-related sickness absence (£6243.60 per manager), we estimate SE for cost savings:

$$SE_{\text{cost savings}} = \left(\frac{SE_{\text{reduction}}}{\text{Reduction in Sick Leave}}\right) \times \text{Cost Savings} \tag{21}$$

$$SE_{\text{cost savings}} = \left(\frac{0.33\%}{0.28\%}\right) \times 6243.60 \tag{22}$$

$$SE_{\text{cost savings}} = (1.178) \times 6243.60 \tag{23}$$

$$SE_{\text{cost savings}} \approx 735.77 \tag{24}$$

6.2 Confidence Interval (CI) for ROI

$$SE_{\text{ROI}} = \frac{SE_{\text{cost savings}}}{\text{Cost of Intervention}} \tag{25}$$

$$SE_{\text{ROI}} = \frac{735.77}{625.55} = 1.176 \tag{26}$$

Using the 95% confidence interval formula:

$$CI = ROI \pm (1.96 \times SE_{\text{ROI}}) \tag{27}$$

$$CI = (7.67, 12.29) \tag{28}$$

7 Paper-Noben et al., 2014 Reference 17

7.1 ROI Formula

The Return on Investment (ROI) formula is given by:

$$ROI = \frac{\text{Cost Savings} - \text{Intervention Cost}}{\text{Intervention Cost}}$$

7.2 ROI Calculation Using the Provided Median ICER

7.3 Occupational Physician (OP) Condition

ICER = €5049 savings per responder
Intervention Cost = €1266 per participant
$\text{ROI}_{\text{OP}} = \frac{5049}{1266} = 3.99$

7.4 E-Mental Health (E-MH) Condition

ICER = €4054 added cost per responder
Intervention Cost = €1375 per participant
$\text{ROI}_{\text{E-MH}} = \frac{-4054}{1375} = -2.95$

8 Paper-El-Mallakh et al., 2014 Reference 18

This document estimates the standard error (SE) for the return on investment (ROI) calculation based on cost and savings estimates.

8.1 Given Data

$$\text{Total Cost} = \$2,430,128 \tag{29}$$

$$\text{Total Savings} = \$3,455,025 \tag{30}$$

$$\text{Net Savings} = \text{Total Savings} - \text{Total Cost} = 3,455,025 - 2,430,128 = \$1,024,897 \tag{31}$$

$$ROI = \frac{\text{Net Savings}}{\text{Total Cost}} = \frac{1,024,897}{2,430,128} = 42.2\% \tag{32}$$

8.2 Standard Error Estimation

Assuming a coefficient of variation (CV) of 25%, we estimate the standard deviation:

$$SD_{\text{cost}} = \text{Total Cost} \times CV = 2,430,128 \times 0.25 = 607,532 \tag{33}$$

$$SD_{\text{savings}} = \text{Total Savings} \times CV = 3,455,025 \times 0.25 = 863,756 \tag{34}$$

The standard error of ROI is given by:

$$SE_{\text{ROI}} = \sqrt{\left(\frac{SD_{\text{savings}}}{\text{Total Cost}}\right)^2 + \left(\frac{SD_{\text{cost}} \times ROI}{\text{Total Cost}}\right)^2} \tag{35}$$

$$SE_{\text{ROI}} = \sqrt{\left(\frac{863,756}{2,430,128}\right)^2 + \left(\frac{607,532 \times 0.422}{2,430,128}\right)^2} \approx 21.2\% \tag{36}$$

8.3 Confidence Interval

Using a 95% confidence interval (CI):

$$CI = ROI \pm 1.96 \times SE_{\text{ROI}} \tag{37}$$

9 Paper-Ward et al., 2023 Reference 22

To calculate the average ROI, we take the estimated salary savings and intervention costs for different salary levels and compute their respective ROIs.

Formula for ROI (%)

$$ROI(\%) = \left(\frac{\text{Salary Savings} - \text{Intervention Cost}}{\text{Intervention Cost}}\right) \times 100 \tag{38}$$

Assuming an intervention cost of $450 per employee, let's compute the ROI for each salary level:

**Annual Salary ($)**	**Salary Savings (6 months) ($, 95% CI)**	**ROI Calculation (%)**
15,080	1,053 (393, 1,712)	$\frac{1053-450}{450} \times 100 = 134\%$
25,000	1,745 (652, 2,839)	$\frac{1745-450}{450} \times 100 = 288\%$
50,000	3,491 (1,305, 5,677)	$\frac{3491-450}{450} \times 100 = 675\%$
63,179	4,411 (1,649, 7,174)	$\frac{4411-450}{450} \times 100 = 880\%$
75,000	5,236 (1,957, 8,516)	$\frac{5236-450}{450} \times 100 = 1064\%$
100,000	6,982 (2,609, 11,355)	$\frac{6982-450}{450} \times 100 = 1452\%$
125,000	8,727 (3,262, 14,193)	$\frac{8727-450}{450} \times 100 = 1839\%$
150,000	10,473 (3,914, 17,032)	$\frac{10473-450}{450} \times 100 = 2227\%$
200,000	13,964 (5,219, 22,709)	$\frac{13964-450}{450} \times 100 = 3003\%$

Table 3: ROI Calculation for Different Salary Levels

10 Paper-Roesner et al., 2024 Reference 23

From the data:

- **Total cost savings**: $14,078,015 - 7,406,136 = 6,671,879$ EUR
- **Program cost per person**: 550 EUR
- **Number of employees**: $1,000$
- **Total program cost**: $550 \times 1,000 = 550,000$ EUR

$$ROI = \frac{6,671,879}{550,000} \times 100 = 1,213.06\% \tag{39}$$

10.1 Standard Error (SE) of Cost Savings

The standard error of savings is estimated from the reported 95% quantiles of HIE-related costs:

- **Upper 95% quantile (without PSP)**: $79,443$ EUR
- **Upper 95% quantile (with PSP)**: $68,222$ EUR
- **Difference (cost savings variability)**: $79,443 - 68,222 = 11,221$ EUR
- **Estimated SE of savings**:

$$SE_{\text{Savings}} = \frac{11,221}{1.96} = 5,725 \text{ EUR} \tag{40}$$

10.2 Standard Error (SE) of ROI

The standard error of ROI is computed as:

$$SE_{\text{ROI}} = \frac{SE_{\text{Savings}}}{\text{Program Cost}} \times 100 \tag{41}$$

$$SE_{\text{ROI}} = \frac{5,725}{550,000} \times 100 = 1.04\% \tag{42}$$

10.3 95% Confidence Interval (CI) for ROI

The confidence interval for ROI is given by:

$$CI = ROI \pm 1.96 \times SE_{\text{ROI}} \tag{43}$$

$$CI = 1,213.06\% \pm (1.96 \times 1.04\%) \tag{44}$$

$$CI = 1,213.06\% \pm 2.04\% \tag{45}$$

$$CI = (1,211.02\%, 1,215.10\%) \tag{46}$$
